# Seeking consensus on the domestication concept

**DOI:** 10.1098/rstb.2024.0188

**Published:** 2025-05-15

**Authors:** Robert N. Spengler, Li Tang, Marta Dal Corso, Rosalind Emma Gillis, Hugo Rafael Oliveira, Basira Mir Makhamad

**Affiliations:** ^1^ Domestication and Anthropogenic Evolution Research Group, Max Planck Institute for Geoanthropology, 07745 Jena, Germany; ^2^ Department of Geosciences, Università degli Studi di Padova, 35131 Padova, Italy; ^3^ Referat Naturwissenschaften, Deutsches Archäologisches Institut, 14199 Berlin, Germany; ^4^ ICArEHB, University of Algarve, 8005-139 Faro, Portugal

**Keywords:** domestication, origins of agriculture, cultivation, archaeology

## Abstract

The domestication of plants and animals permitted the development of cities and social hierarchies, as well as fostering cultural changes that ultimately led humanity into the modern world. Despite the importance of this set of related evolutionary phenomena, scholars have not reached a consensus on what the earliest steps in the domestication process looked like, how long the seminal portions of the process took to unfold, or whether humans played a conscious role in parts or all of it. Likewise, many scholars find it difficult to disentangle the cultural processes of cultivation from the biological processes of domestication. Over the past decade, the prevailing views among scholars have begun to shift towards unconscious and protracted models of early domestication; however, the nomenclature used to discuss these changes has been stagnant. Discussions of early domestication remain bound up in prevailing definitions and preconceived ideas of what the process looked like. In this paper, we seek to break down definitions of domestication and to construct a definition that serves equal utility regardless of the views that researchers hold about the process.

This article is part of the theme issue ‘Unravelling domestication: multi-disciplinary perspectives on human and non-human relationships in the past, present and future’.

## 1. Introduction

Charles Reed once stated that ‘Domestication is not a clean-cut concept, and the word is difficult to define. I have become lost in this semantic bog before, and so avoid the morass now’ [1, p. 19].

In this article, we intend to dive into Reed’s bog and seek a domestication concept that is applicable across the sciences, unbound by preconceived ideas or researcher biases. Domestication is one of the most important biological phenomena to pull humanity into the culturally modern world, providing high-yielding crops and food surplus [2]. However, there has never been a consensus among scholars regarding what domestication is, how it occurs or even what organisms are domesticated. This confusion has grown over the past few years, as domestication scholars are increasingly shifting favour towards ecological models of early plant and animal domestication. In 2010 [3], Fuller declared that a paradigm shift had occured in the field, following the resurrection of ideas earlier laid out by Rindos [4]. This shift is marked by a retreat from models of domestication that rely on active, conscious and rapid domestication (core-area one-event model [5]) and growth in popularity of models that de-emphasize human innovation in favour of protracted and unconscious processes occurring along multiple parallel lineages (variously called the protracted-autonomous model [5], coevolutionary model [4,6] or a rindosian approach [7]). This new (or resurrected) approach to domestication theory is especially hard to syncronize with popular definitions of what domestication is and how the word should be applied. In this paper, we seek a domestication concept that is usable regardless of the approach that a scholar embraces. Our goal is to construct a simple definition that can be used by scholars despite their views about the level of human intentionality, time durations involved or differences between pathways to domestication.

Language dictates concepts, especially in the sciences, where preconceived ideas, value judgements, familiarity biases and researchers’ personal morals and opinions can direct research agendas and interpretations. The awareness of this process is variously called the Sapir–Whorf Hypothesis, Linguistic Relativity Hypothesis or Linguistic Determinism, all of which build on the philosophy of Wittgenstein [8]. The ways scholars use the word ‘domestication’ tend to favour conscious and rapid models and rational explanations to the widely asked question of why humans did it (the push and pull arguments). This is most notable when domestication is used in an active sense; i.e. they domesticated that crop, as opposed to the passive; i.e. that crop evolved domestication traits. In this way, the domestication concept, as it currently stands, biases interpretations towards the Core-Area One-Event Model and humanist perspectives. A scientific definition should seek to avoid interpretation biases and should not direct research in favour of any particular scholar’s views or that of a group of scholars. There have been many scholarly attempts at defining a domestication concept (e.g. [9–15]; see also the electronic supplementary material and table 1), and we seek to build on these previous attempts, taking into account changing perceptions in scholarship. As O’Connor [11, p. 149] noted, scholars are still ‘struggling to find a satisfactory definition’, and Russell [27, p. 286] emphasized that ‘It has proven remarkably difficult to formulate a satisfactory definition’. In this paper, we seek to deconstruct published definitions and propose an encompassing definition that can be equally applied regardless of preconceived ideas of what the earliest steps in the domestication process looked like. Two recently published debates illustrate why a clear definition is essential; they involve the concepts of anti-domestication, landscape domestication and the failure-to-domesticate paradox.

**Table 1.** A list of published definitions and the caveats that each author(s) includes in their definition. Each full definition is quoted in the electronic supplementary material.

reference	nine key caveats^a^								
Galton 1865 [16, p. 138]						X			X
Zeuner 1963 [17, p. 9]			X						
Bökönyi 1969 [15, p. 219]		X	X			X	X	X	
Hale 1969 [18, p. 22]								X	X
Belyaev 1978 [19, p. 307]						X			
Belyaev 1978 [19, p. 308]			X			X			
Ducos 1978 [20, p. 54]			X				X	X	
Price 1984 [21, p. 1]					X	X			
Price 1984 [21, p. 1]		X							
Harlan 1992 [22, p. 175]								X	
Clutton-Brock 1994 [23, p. 26]			X			X	X	X	
Ladizinsky 1998 [24, p. 7]	X	X		X					
Clutton-Brock 1999 [25, p. 30]							X		
Clutton-Brock 1999 [25, p. 31]								X	
Diamond 2002 [26, p. 700]			X						
Russell 2002 [27, p. 286]		X							
Balon 2004 [28, p. 2]						X	X	X	
Gepts 2004 [29, p. 2]			X			X			
Gepts 2004 [29, p. 6]			X	X					
Gepts 2004[29, p. 7]						X			
Dobney & Larson 2006 [30, p. 261]									
Dobney & Larson 2006 [30, p. 268]							X		
Zeder *et al*. 2006 [31, p. 139]	X	X							
Bilio 2007 [32, p. 7]						X			
Driscoll *et al*. 2009 [33, p. 9972]					X				
Driscoll *et al*. 2009 [33, p. 9972]						X			
Trut *et al*. 2009 [34, p. 349]						X	X		
Cieslak *et al*. 2011 [35, p. 886]					X				
Cieslak *et al*. 2011 [35, p. 886]						X			X
Cieslak *et al*. 2011 [35, p. 894]					X	X			
Lee *et al*. 2011 [36, p. 3]				X					
Harris 2012 [37, p. 48]							X		
Meyer *et al*. 2012 [38, p. 29]	X					X			
Fuller & Hildebrand 2013 [39, pp. 507, 508−509]									
Fuller *et al*. 2014 [40, pp. 6147−6148]		X							X
Larson *et al*. 2014 [41, p. 6140]									X
Larson *et al*. 2014: [41, p. 6141]					X				
Teletchea & Fontaine 2014 [42, p. 187]							X	X	
Zeder 2015 [43, p. 3191]				X					
Teletchea 2017 [44, p. 6]					X				X
Allaby *et al*. 2017 [45, p. 17]			X	X					
Hunter 2018 [46, p. 201]		X		X					
Hunter 2018 [46, p. 205]				X					
Stépanoff & Vigne 2018 [47, p. 20]			X	X					
McHugo *et al*. 2019 [48, p. 98]						X			
Purugganan 2022 [12, p. 664]							X		
Purugganan 2022 [12, p. 669]	X		X	X			X		
*National Geographic*			X				X		
*Merriam-Webster*						X			
*Cambridge Dictionary*			X						
*Britannica*	X						X	X	

^a^ Caveats: 1, Does the trait have to be heritable? 2, Does the evolution of the trait have to occur on a population scale? 3, Does the trait have to benefit humanity? 4, Does the trait have to benefit the other organism? 5, Are their only specific traits that should be included? 6, Do we only include traits that evolve through intentional selection, i.e. breeding? 7, Does one organism have to control the others? 8, Does the definition have to involve a concept of property or ownership? 9, Is there a mandatory time variable?

### (a) Anti-domestication, landscape domestication and the failure-to-domesticate paradox

To emphasize the need for a clear and agreed-upon definition, we will summarize two debates that unfolded in articles published in 2021–2022 [12,49–51]. The first is in response to an attempt at (re)defining domestication by Purugganan [12], whereas he limits the definition to organisms tied into an ‘agriculture-type mutualism’ [52]. Purugganan [12] was particularly interested in compiling a definition that included agriculture-type mutualistic relationships between certain ants and fungi or aphids as well as certain fish and algae or shrimps (as a few examples). In doing so, he constructed a definition based on mutualistic relationships where one organism protected, supplied resources to, and controlled another and in turn either ate or extracted resources from the controlled member of the relationship (see electronic supplementary material). Clement responded to this article by claiming that ‘control’ of one organism over another is not a necessary attribute of domestication [49]. He suggested taking a closer look at the way Rindos used the concept of domestication [4]. Purugganan [53] rebutted, further emphasizing that his specific definition limits the parameters of the domestication concept to evolution resulting from a specific type of mutualism. This debate echoes much older discussions of the concept of domestication [27]. Interestingly, Clement *et al*. [54] had also campaigned their own domestication concept, surrounding what they call ‘landscape domestication’ [55–58]. Clement *et al*. [59] argued that we should count all ecological impacts of humans on their landscape as domestication; specifically they see shifts in the population structure of the Amazonian forests as indicative of a domesticated landscape. Following their concept, human presence or activity of any kind constitutes domestication of the landscape. In their paper, 'The domestication of Amazonia before European conquest', they remove the link between domestication and evolution and instead link domestication to human presence, claiming that people were present several millennia ago, so the Amazon forests were domesticated at that time [54,59]. They disclose at the onset that they developed the concept of a domesticated Amazon with the aim of combatting antiquated perceptions of a pristine forest. There does not appear to be a consensus on what landscape domestication really means, and all applications appear to be tied to a moralistic exclamation that ancient people were as populous and culturally ‘developed’ as other people [60].

Landscape domestication seems to be yet another synonym for the terms that have recently arisen to describe human/environmental interactions, such as Niche Construction Theory, Ecological Engineering or Anthropocene (for other examples of terms applied to the same concept, see the discussion by Spengler [61]). All humans construct niches, they alter vegetation communities, and they change landscapes; while it is a worthwhile goal for archaeologists to identify the ecological legacies of these impacts, a new concept or theory is not needed to define them. It would be more meaningful to clarify how humans as ecosystem engineers or niche constructors are more impactful than non-human primates constructing ‘monkey orchards’ [4,7] or elephants creating trails of fruit trees [62], and certain avian species, such as corvids and parrots, altering vegetation communities around their roosts. All humans clear openings in forests and carry foraged fruits into that opening, creating a new vegetation community at that location; Anderson [63] recognized this with his Dump Heap Hypothesis (an idea that found its origins in the writings of Darwin [64]).

The domesticated landscapes concept has also been campaigned as part of a new movement in domestication theory called ‘anti-domestication’. The anti-domestication concept also arises out of studies in the Amazon, which claim that ‘an Amerindian notion of sharing territory with other living beings and their different demands’ would not lead to domestication [65]. This is a direct resurrection of a Rousseauean view of humans existing as part of nature and not affecting their surrounding ecosystems, and is tied into deep-seated ideas of conscious domestication and intentional rational drivers of innovation. The term stands in direct contrast to the concept of landscape domestication, but proponents of these views seem to overlook the contradiction and latch onto both concepts, seemingly on ideological grounds. Even more perplexingly, these scholars often claim to be promoting de-colonialist ideals, but they march under the colonialist banner of the noble savage. Ironically, all of these scholars are actively embracing a concept of domestication that relies on human innovation and mastery over nature, but they differ in their judgements regarding whether domesticating or altering an ecosystem is a good or bad act. Ancient peoples of the Amazon did drive the evolution of domestication traits in many tree species, although a dearth of archaeobotanical research has shrouded the antiquity of these evolutionary processes. For example, Clement *et al*. [54] note that there is a lack of archaeobotanical data to discuss the domestication of the peach palm, so they speculate that the magnitude of change under domestication indicates that it must have been domesticated 10 000 years ago. Applying the same logical fallacy to the pineapple, they claim that it was domesticated 6000 years ago [54]. Despite a complete lack of data, these claims are widely cited and propagated [66,67]. This example further illustrates the need for standards in both the use of terminology and also the criteria used to study domestication. Recent research is showing that archaeobotanical material can be preserved in the Amazon [66], illustrating that there is a great potential to understand domestication processes through scientific means.

Archaeologists have long lamented what Stahl [68, p. 221] calls the ‘paradox of animal non-domestication’, the fact that certain peoples in certain parts of the world did not domesticate animals, such as in Africa and the Amazon. This fact clearly frustrates anthropologists who equate domesticating with intelligence, and Stahl [68, p. 221] seeks to break down the ‘failure-to-domesticate’ view by claiming that some people had a different ‘perspectival ontology’ that made them not want to domesticate animals. Stahl [68] is pulling his argument from Hugh-Jones [69, p. 246], who likewise combats what he calls the ‘failure-to-domesticate’ paradox by stating that for these ancient peoples ‘true domestication is probably something more inconceivable than impossible’. All of these discussions assume that domestication is a process relying on intelligence, whereas unconscious models of domestication build on the view that it was inconceivable to all ancient peoples. Their argument is, if a people see themselves as a peaceful part of nature, they would not wish to control an organism through domestication—this is, of course, also a direct harkening back to a Rousseauean dove argument. Providing a slightly different answer to the failure-to-domesticate paradox, Diamond [26] responded by stating that not all animals are domesticatable, and claimed that the African continent was depauperate in domesticatable animals (a nonsensical statement when we recognize that wild relatives of most of the domesticated species from Eurasia existed in Africa). The anti-domestication movement has grown in popularity, seemingly owing to a moralistic linking of the idea with anti-colonialism or non-colonialism [70]. Although these scholars fail to point out that Europe itself falls into the failure-to-domesticate paradox, as the majority of humanity’s domesticated animals evolved in Asia.

The anti-domestication discussions have been picked up by other scholars and drawn into the decolonialization movement in archaeology. This is well evidenced by Bogaard *et al*. [50], who claim that there is a colonialist legacy in the way scholars use the term domestication, notably concepts of domestication that rely on active, conscious human innovation and mastery over nature. Bogaard *et al*. [50] were criticized by Abbo & Gopher [51], who implied that they embraced the decolonialization movement more as a marketing tactic. Abbo & Gopher are correct in stating that Bogaard *et al*. make no real effort to explain what the colonial legacy is, nor do they give examples of the immoral uses that they claim exist. If, indeed, the term ‘domestication’ is a legacy of imperialism, then there is greater need to re-evaluate the definition, making our present article even more important. However, both the claim for a need to decolonialize the term and the entire anti-domestication movement assume a correlation between a degree of cultural complexity (or development) and the number of domesticated species cultivated. Much of the above-mentioned literature conflates early domestication with early cultivation; these are linked but different concepts—a people can cultivate a plant and that plant may not evolve domestication traits—likewise, we argue in this paper that domestication traits can evolve without cultivation.

Responding to the critique by Abbo & Gopher [51], Fuller *et al*. [71, p. 2) claim that ‘Bogaard et al. [50] point out that language used to describe domestication has its roots in nineteenth century European thinking. This historical background means that revised and inclusive definitions of domestication are needed’. Indeed, we all agree that revised and inclusive definitions are needed, but Bogaard *et al*. [50, p. 60] base their claim of a colonialist legacy in the domestication concept on one quote from Buffon, whereas he defines domestication with the caveat that the domesticated organism has to serve a use for humans. However, prior to the paradigm shift in 2009, nearly all domestication scholars held some form of this view assuming human innovation. While Buffon was an early evolutionary thinker, it is true that his ideas of evolution looked different from those of Darwin or early members of the Darwinian school. Bogaard *et al*. [50] are accurate in pointing out that Darwin favoured unconscious arguments for early domestication and did not discuss human ‘agency’ over nature. Likewise, essentially, all of the early Darwinian scholars in the domestication debates (e.g. Vavilov, de Candolle, Haeckel, Darlington, Anderson and Sauer) shared a view more in line with the Protracted-Autonomous Model. Bogaard *et al*. [50, p. 60] state that ‘For Buffon, mirroring wild animals created by God, domestic animals are quasi “creations” of human labour. They have been withdrawn from the rules of nature and made “unnatural”: “Man changes the natural state of animals by forcing them to obey and serve him for his use”' [72]. While Buffon historians have debated over his religious beliefs, it is generally accepted that he was agnostic, and indeed he was forcibly ordered to retract his writings by ecclesiastic authorities [72–74]. Regarding his views on domestication, we can consult Buffon directly, who stated: ‘The grain with which man makes his bread is not a gift of Nature but the great, useful fruit of his researches and his intelligence in the first of the arts’ [75, p. 130]. While he did adhere to a conscious model of domestication, to berate one of the greatest scientists of the eighteenth century for being religious, when he was persecuted in his lifetime for being atheist, seems rather cruel.

The concept of domestication as representing (i) a human mastery over nature, (ii) a conscious active event, (iii) human ‘agency’ and (iv) models that focus on rational decisions, are all ideas prominent in thought among many different peoples around the world and through time (humans, essentially by default, are anthropocentric). However, these humanist ideals rose in popularity in the domestication debates following Sahlins’s [76] depictions of the original affluent society. If any one event or group of scholars is to be credited with the resurrection of ‘humanist’ or romanticist concepts of domestication and human mastery over nature (presumably what Bogaard *et al*. actually mean when they say colonialist) it is the attendees of the *Man the hunter* conference in 1966, who saw a moralistic need to emphasize human dominance and superiority [77]. Sahlins and the other anthropologists involved in this revisionist perspective were openly drawing on a belief that they needed to empower pre-farming peoples and that views of farming being more adaptive than foraging were socially problematic. Their resurrection of a Biblical Eden or a community of ancient Rousseauean doves directly implied that farming represented a destruction of the harmonious state that foraging communities lived in (for discussions of the reasons why Sahlins exaggerated his data, see [78–80]).

The idea of domestication as both an innovation and a destructive force upon nature and humanity (the great Faustian Bargain) grew in popularity among archaeologists through the 1980s and 1990s. The Push arguments were developed based on the premise that farming is maladaptive and that humans must have been forced to invent domesticated plants by some extreme external driver. The domestication concept that Bogaard *et al*. [50] are retaliating against can more easily be attributed to anthropologists of the late twentieth century. The views resurrected by archaeologists from the 1980s until the paradigm shift of 2009 are directly tied to humanist ideals of the Counter Enlightenment or Romanticist movement. The archaeology community of the 1980s and 1990s so fervently embraced the ideas of domestication as the greatest of the human innovations and discussed active human ‘agency’ in the process that when Rindos [4] presented a narrative that resurrected earlier concepts from Darwin, he was essentially run out of academia [31]. Bogaard *et al*. [50] seem to completely invert this. The fact that the prevailing moral agenda of archaeologists has continually swung like a pendulum illustrates how important it is to have definitions divorced from moral biases.

The connection of the term ‘domestication’ with a colonialist legacy, and the rise in popularity of concepts of anti-domestication, landscape domestication and the failure-to-domesticate paradox represent attempts at using the term to promote ideologies. Ironically, in all of these cases, the researchers are criticizing other researchers on ideological grounds, who, in turn, were pushing their own moral views, leading to the chains of circular rebuttal articles discussed above. Landscape domestication scholars seek to empower ancient peoples by arguing that they drastically altered local ecologies and burned the jungles; anti-domestication proponents seek to empower ancient peoples by arguing that they chose not to alter local ecologies or burn the forests owing to their unique ontology. Post-processual archaeologists sought to empower ancient peoples by arguing that domestication resulted from human innovation; decolonialist proponents claim that domestication models based on human innovation are a legacy of an Eurocentric era. To the contrary, Enlightenment scholars (while holding a wide range of views) generally envisioned unconscious models of domestication, whereas Counter Enlightenment scholars imaged great human accomplishments in prehistory. It is essential that we develop a definition that removes all ideological trappings and does not rest on preconceived ideas of what the earliest steps in the domestication process looked like.

### (b) A morass of linked terms

As with anti-domestication, many other terms and ideas in scholarship rely on a specific domestication concept. For example, the increasingly prominent use of the term ‘de-domestication’ (e.g. [81,82]; Gamborg *et al*. [82] use a different definition for de-domestication, which is synonymous with the term re-wilding) would seem to imply that evolution is reversible, which contradicts basic principles of evolutionary biology (according to the Hardy–Weinberg equilibrium, evolution can neither reverse nor become stationary). Other terms that have been criticized elsewhere include ‘incipiently domesticated’ and ‘semi-domesticated’ [54]. Meyer *et al*. [38, p. 29] promote the concept of ‘semi-domesticated’ as a middle ground between what they call ‘undomesticated’ and domesticated species (also see [83, p. 905]). Fuller *et al*. [40, p. 6148] state that a ‘domestication episode’ is ‘the period in which key domestication syndrome traits underwent directional change and approached fixation within cultivated populations: these traits are now normally shared among all populations of a specific crop’. Clement *et al*. [54, p. 74] point out that ‘the term “proto-domesticate” is often used, but *protós* is Greek for first, leading to definitions such as “original” and “primitive” (as in “first order”); since domestication is a process and the domesticated population is the result, the domesticate is not primitive but derived. Hence, the term should be avoided’. Following this reasoning, most of these terms are teleological, as articulated by Bates [9, p. 1], stating: ‘Terms like “proto” should be abandoned, as they imply directedness, terms with modern burden like “cultigen” likewise should be avoided unless there is due reason to use them’.

In addition to impacting academic squabbles, having a clear definition for what domestication is has practical implications, including in conservation initiatives. It has long been recognized that species concepts can impact conservation efforts, as a subspecies or variety is far less likely to attract conservation attention than an endangered species [84,85]. Likewise, funders are less likely to fund conservation for domesticated species as there are negative connotations attached to those organisms, but if scholars use a more open-ended definition of domestication (as we promote below), then the statement that humans are rapidly domesticating all organisms on the planet may further garnish attention.

## 2. Deconstructing definitions

Most definitions of domestication are modular, with scholars linking together caveats that build parameters for their definitions. In this way, they decide which organisms to include and which to eliminate from their constructed category of ‘domesticated’. We pull out nine of the most prominent caveats that scholars use when constructing these modular definitions (table 1 and electronic supplementary material), two of which we ultimately choose to accept in our proposal for an inclusive definition.

### (a) Heritability

There are three ways that organisms adapt to their environment: through real time, *Regulatory*; lifetime scale, *Developmental*; and generational scale, *Evolutionary* adaptations. While developmental responses can be evolutionarily significant, they are not directly passed onto the next generation [86]. Domestication is an evolutionary process, and therefore, developmental responses in plants and animals should not be included in the domestication concept, especially since a suite of poorly defined terms referring to human-induced developmental responses already exists: i.e. taming, learned behavior, learning, conditioning, training, habituating, Pavlovian response, etc. Likewise, the ability to express variability in developmental responses is called plasticity [87–90], and there are already a range of terms used to refer to developmental plasticity in relationship to humans: weedy, anthropophilic, adaptable, tameable, domesticatable, invasive, etc. Taming is a non-heritable change in the rates of release of stress hormones among animals owing to proximity to humans; when it becomes heritable, this change is called domestication (commensal or parasitism on humans) as identified in organisms as wide-ranging as the birds at your birdfeeder or the mice in your walls [88,91–93]. Plasticity itself is an evolvable trait, and organisms in the wild tend to evolve greater ranges of plasticity or wider reaction norms in response to dynamic environments [87,94,95]. As anthropogenic environments are highly dynamic, the evolution of weeds, commensals and pests is usually accompanied by an increased range of developmental plasticity. Likewise, organisms with a high range of developmental plasticity are exapted to these niches and more readily evolve domestication traits (see discussion in [61]).

### (b) Population-scale process

While avoiding debates over whether natural selection acts on populations, species or genes [96,97], the process of evolutionary change unfolds on a population scale. Consequently, domestication is a population-scale process; this is a concept that often trips up scholars. A single plant is not domesticated—that plant is part of a domesticated population. It is essential to keep this clear in scientific discussions of domestication, as a single archaeobotanical rachis is not an indication of domestication, but rather a high prevalence of tough rachises across a population (a population could be a cultivated field, a farmer’s saved seeds, a landrace, ecotype or an archaeobotanical assemblage) indicates the domesticated classification of that full population even if some members of that population resemble wild morphs. Spengler [98] recently suggested that archaeobotanists stop thinking about tough versus brittle rachises as a varietal dichotomy, but rather as two morphs in a population that, in the wild, is dimorphic in its seed-dispersal system. Dimorphic seed-dispersal systems readily shift the frequencies of each morph in the wild, sometimes in response to rather minor ecological changes.

### (c) Benefits to humans

Many scholars have formulated arguments that domestication is only domestication if the traits that evolve benefit humans [45,99,100]. Obviously, any definition in science that relies on an individual value judgement is going to invite bias, but this caveat is particularly problematic as it would eliminate commensalism from the concept. Evolution of certain crops, such as rye and oats, likely first started as weeds in cultivated fields, likewise, many domesticated animals, such as cats and dogs, likely started as commensals or dump-heap foragers [7]. Adding the human-value caveat suggests that all commensal domesticates were not domesticated until humans realized that they could benefit from those organisms, regardless of those organisms’ evolutionary changes. Presumably, these scholars have added that caveat in order to eliminate mice, rats, cockroaches and other commensals/parasites from their definition, but if humans began more regularly eating rats or using them as pets or for lab experiments, would this suddenly alter their domestication status? Likewise, if humans gain little benefit from feral cats and dogs, do they still count as domesticated under this definition? Furthermore, would animals that evolved smaller body sizes owing to heavy hunting or predation pressure still count as domesticated, given that the specific evolutionary change does not benefit humanity? Following the same reasoning, are animals on game preserves that evolve smaller horns or antlers owing to heavy trophy hunting domesticated even though the hunters do not favour such changes?

The distinction between the evolution of weeds and that of crops is probably most blurred in the case of crop mimicry, ‘the ability to escape human removal by physically resembling the crop in which weeds grow’ [101, p. 305]. These weeds adapt to the anthropogenic cultural practices of cultivation in essentially the same way that crops evolved, and in some cases, the mimics eventually became crops. Rather than thinking about these weeds mimicking domesticated crops, it is equally as appropriate to think about them evolving domestication traits.

The claim that an organism is only domesticated if humans appreciate and benefit from it is unnecessarily anthropocentric. Purugganan [12], as discussed above, recently attempted to take this caveat and make it less anthropocentric by stating that the term domestication should only apply to organisms that evolve in a mutualism to benefit (or be controlled by) one member of the mutualism (electronic supplementary material, table S1). His new definition is expanded to include ants that are in an agriculture-type mutualism with fungi. Purugganan emphasizes that his definition is restricted to organisms that evolve specifically to benefit humans (or non-human domesticators, such as certain ants), stating that the term domesticated is often ‘mistakenly applied in relation to entities as diverse as commensal species, weeds, transposable elements, and even humans’ [12, p. 9]. All symbiotic relationships, by definition, benefit at least one member. Likewise, many mutualisms benefit one member more than the other, but are not part of an agriculture-type mutualism, for example, did angiosperms domesticate pollinators and seed-dispersers to service them? Many angiosperms provide nectar, sugary fruits, edible foliage or shelter for pollinators or seed-dispersers; does this mean that species, such as *Acacia* spp., with swollen thorn domiciles [102], domesticated the ants that are locked into an obligate mutualisms with them?

### (d) Benefits to other organisms

Another way to avoid the overt anthropocentricism of many definitions is to focus on the role of or value for the other animal or plant in the relationship. Often the scholars who add this caveat discuss the ways humans enhance the survival or competitiveness of plants and animals [39,103]. Arguably, the increased adaptive advantage to the other organism does not require stating, as, from an adaptationist perspective, the other organism will not evolve traits that do not present it with an advantage. Often these scholars seem to see their focus on what they sometimes call the ‘agency’ of plants as a moral retaliation against a long history of anthropocentric concepts of domestication (for an extreme case, see Wang [104], who laments that the agency of ceramic pots has been neglected), combatting what they see as ‘Anthropocentric bias and ignorance’ (Blattner *et al*. [105, p. 1]. In some ways, this view is an elaboration of Rindos’s question: who domesticated whom, the grass or the human? While Rindos formulated this question, it builds on earlier mutualism discussions in domestication [22,63,106]. This question has also been popularized by Pollan [107], Harari [108] and Scott [109]. Rindos [4, p. 93] ultimately answers the question by saying: ‘we might equally well describe the evolution of domesticated plants by saying that the plants chose humans to protect and disseminate them, or that the plant adapted by using humans to increase their own fitness’. While Rindos’s answer is accurate, it is more appropriate to say that in a mutualistic relationship, both organisms coevolve in response to the other, often locking a tighter mutualism in place—in some cases, leading to an obligate mutualism. It is easiest to view the process from the human perspective, but if we think about it from the plant perspective, maize, wheat or soya beans have manipulated humans into changing nearly every ecosystem on Earth in favour of their population expansions.

While both organisms in a mutualism benefit, it is not always easy to identify evolutionary changes directly resulting from that mutualism in both organisms. Often scholars add the caveat of ‘coevolution’ to their definitions of domestication, following Rindos [4]. However, scholars have struggled to clearly identify phenotypic traits in humans that have evolved as a response to the mutualism with grass. In fact, Spengler [61] recently pointed out that the main case studies in gene–culture–coevolution literature have all been challenged or disproven over the past few years. Arguably, one of the only remaining clear examples of humans evolving to better adapt to this mutualism is an increase in amylase production in the saliva of some (not all) farming populations [110]. Additionally, the coevolution argument does not apply to all domesticated organisms; for example, have humans coevolved with chocolate or cats?

### (e) Limiting the range of anthropogenic evolution

Domestication scholars often use phrases or terms that limit what parts of the range of anthropogenic evolution count as domestication. For example, Allaby *et al*. [45, p. 2] discuss ‘true domestication traits’, standing in contrast to the wide array of other traits that evolved under human cultivation, which they refer to as ‘diversification or crop improvement’ traits. Many scholars discuss a terminus to the domestication process, claiming that domestication only encompasses the earliest trait to evolve in each crop [45]. Needless to say, these scholars are all archaeologists or ancient geneticists, and the drive to continually identify ‘the oldest’ or ‘the first’ of everything seems to be dictating this scholarly trend. Some scholars make arbitrary divisions along the evolutionary spectrum to refer to ‘fully domesticated’ organisms. An extreme example of this comes from Scott [109], who claims that only an evolutionary ‘super floral basket case’ should count as domesticated—limiting the term ‘domesticated’ to only obligate domesticates. In this way, Scott does not consider pigs, dogs or cats as being fully domesticated, seeing that some members of these clades could survive without human intervention.

Another definition that limits the range of evolutionary traits that can be called domestication comes from Larson & Fuller ([111]; see electronic supplementary material, table S1), who insert the caveat of the process taking a long time to unfold. While many of the authors of that paper have presented rather strong arguments for protracted models for early cereal crops, they have fallen far short of illustrating it for all crops. Likewise, some intriguing arguments exist for the possibility of rather rapid domestication in certain cases [112]. Notably, many long-generation perennials were presumably domesticated through hybridization, suggesting that they underwent a transition to ‘domesticated’ in one rapid event, if we assume a clonal population of genets counts as a domesticated population [113]. Regardless of whether most crops evolved their first domestication traits rapidly or over a long period of time, a proper definition should be able to accommodate all of these scholarly theories. Given that domestication is evolution, it is best to consider domestication as a continuing and inevitable process. The claim that the evolution of some domestication traits are crop improvements and others are actual domestication is, again, the insertion of researcher biases and judgements. For the adaptability of the plant, the evolution of glyphosate resistance, variegated foliage, doubled petals or parthenogenic morphs is just another step in a long process of adaptation to anthropogenic environments. Constructing a teleological framework or claiming that there are ideal domesticated forms is as troubling as claiming that an Australopithecine is an under-evolved human or that the evolution of bipedalism was a true humanizing evolutionary process and all subsequent evolutionary changes were hominid improvements.

### (f) Intentionality

The idea that much, if not all, of the earliest steps in the domestication process were unconscious is not novel, it was well articulated by Darwin (and many of his contemporaries, such as Haeckel), who stated that: ‘unconscious selection is that which follows from men naturally preserving the most valued and destroying the less valued individuals, without a thought for altering the breed; and undoubtedly this process slowly works great changes’ [64, p. 176]. As we have already discussed, arguments for conscious breeding in prehistory were formulated by archaeologists during the second half of the twentieth century and continued to remain prominent in scholarship until 2009, when they started to wane in popularity. It has proven especially difficult for many archaeologists to accept that humans did not invent domesticated crops and that the invention of agriculture was not a response to external variables. It took evolutionary biology more than a century to fully eliminate the ideas of a purposeful evolution—most evolutionists long after Darwin still subscribed to some version of a teleological creed, whether deism, divine intervention (later termed intelligent design), orthogenesis or theistic evolution. Therefore, it is unlikely that all domestication scholars will rapidly switch to the protracted autonomous model, despite the recent increase in popularity of this view. Likewise, most scholars seem to straddle the divide, suggesting that some crops and animals evolved domestication traits unconsciously—both Zeder [114] and Larson & Fuller [111] present arguments for different ‘pathways to domestication’, among which they envision some involving conscious and, therefore, rapid (as the human preview is rarely more than the length of a human generation) ancient breeding. As some examples of organisms with differing perspectives on their domestication pathway, some crops, such as rye, are widely thought to have evolved domestication traits as weeds in cultivated fields [115], and dogs are sometimes depicted as evolving domestication traits through commensalism or as dump-heap foragers [116].

### (g) Post-Modern definitions

Some scholars have constructed species concepts that completely veer away from the core tenets of phenotypic change and evolution; in almost all of these cases, the scholars are assuming active intentional human engagement. In the same way, the anti-domestication movement rests on the assumption that humans who domesticate crops are by some judgement superior to those who do not domesticate crops. Many of the scholars who push for domestication discussions that avoid evolutionary language favour an ideology; often they appear to desire an empowering of some or all ancient peoples by pushing arguments that emphasize human agency over nature. As an extreme case, Wang [104] actively seeks to empower ceramic sherds, suggesting that their agency drove people to consciously domesticate plants so that they had something to cook in the pots. She discusses alternative ontologies that view a pot as having a spirit. Much of the Post-Modern narrative in archaeology can be traced back to the Counter Enlightenment, notably the German Romantics and Naturphilosophie, and transplanted into anthropological theory with phenomenology during the Post-Processual critique. Janik [117, p. 275] epitomizes this humanist agenda in stating that: ‘domestication does not just happen but is carried out by acts of real people and they need to be honored’. It is understandable that so many archaeologists have jumped on this concept, but it remains unclear how aware of the domestication process farmers were.

The domestication concepts that seek to completely divorce the evolutionary variable from domestication largely rose out of the post-processual movement in archaeology [118,119]. In his work with reindeer herders, Ingold stated: ‘the difference between hunting and pastoralism lies not in the particular characteristics of the animals themselves, but in the productive relations that link animals and men’ [118, p. 82]. Ingold intentionally blurs the concepts of domestication and cultivation (or husbandry), and devalues domestication traits as a topic of study. Such scholars sought to remove the systematic and quantitative approaches to domestication studies that had been developed by archaeobotanists and zooarchaeologists, instead favouring an esoteric definition of agency and storytelling unencumbered by scientific data. Some examples include, Graeber & Wengrow’s [78] recent argument for ‘play’ farming, as well as ritual arguments for the origins of domestication. Other scholars have sought to reject the concept of domestication completely, based on the realization that it exists across a continuum [120,121], but, of course, the express goal of all taxonomic systems is to divide populations that exist along a continuum.

### (h) Domestication syndrome

The term ‘domestication syndrome’ was first used by Darwin [64] and resurrected in the 1980s by Hammer [122], with continued use thereafter [29,38,40,123–125]. Its use has been challenged on the grounds that it does not match the medical use of the term ‘syndrome’, such as in Down’s syndrome [126]. However, it is important to remember that Darwin was writing before a gene concept, and had no understanding of the causes of Down’s syndrome (technically the medical community appropriated the term). Despite claims that foxes and dogs do not express a syndrome of domestication [127], some scholars have started linking the changes associated with these species to William’s syndrome—possibly suggesting that in these cases a true medical syndrome did play a major role in the domestication process [128]. Nonetheless, Spengler [98] has pointed out that domestication scholars are using the term in a vernacular or ecological (as opposed to genetic) sense to explain the parallel and convergent evolutionary changes that occurred (also called evolutionary trends), in the same way that island biogeographers use the term Island Syndrome [129] or Insularity Syndrome ([98,130,131]—Spengler [98] suggested replacing both domestication and island syndromes with insularity syndrome).

## 3. Building a scientific definition

### (a) A polyphyletic guild

The terms domesticated, wild, feral, weedy and tamed are all polyphyletic classifications, representing guilds of organisms that are linked by an ecological factor, specifically their relationship to humans. Similar ecological terms for guilds of organisms encompassing biologically unrelated, but ecologically or behaviourally linked, species, include herbivore, omnivore, frugivorous, perennial, bipedal, arboreal, commensal, epiphytic, endozoochoric, pollinator, seed-disperser, etc. In this way, all of these classifications are observational and not predicated upon genetic indicators, although the presence or absence of specific alleles may provide clues regarding which guild a particular species belongs to. For example, a pollinator can be from clades as diverse as: Chiroptera, Aves, Anthophila or Coleoptera. Additionally, the ability to be a pollinator has evolved repeatedly among different lineages within these clades; therefore, the best way to properly classify an organism as a pollinator is to observe it in the act of pollinating. However, as palaeontologists, like archaeobotanists or zooarchaeologists, cannot observe their specimens behaving in their natural habitat, they need to rely on morphological indicators in combination with modern analogy. The process of becoming a domesticated organism is the same as the process of evolving pollination or seed-dispersal traits; in this way, domestication is evolution—the evolution of specific traits associated with adaptation to an anthropogenic ecosystem. Domestication should not be defined on a genetic basis or based upon an indicator allele alone, it is an ecological category, defined by phenotypic traits—morphological and behavioural.

### (b) The species concept framework

In defining the concept of domestication and domesticated, we draw on the legwork already undertaken by taxonomists who have heavily debated the species concept (and every other taxonomic division). As Bates [9, p. 1] emphasized when attempting to define domestication, ‘our choice of “species” definitions carries with it ramifications for our interpretations, and that care needs to be made when handling this challenging classificatory system’. Domestication scholars are actually entering the taxonomy debates late in the game, and many of the issues that need to be raised about the domestication concept have already been aired in debates over the species concept. This is not the place to summarize this large body of literature, as many summaries of the history of debate surrounding the species concept have already been published [132–136]. Wilkins pulled 26 distinct concepts of a species from current scholarly literature, and illustrated the complexity of ongoing discourse [136]. Humans have been classifying populations of organisms for millennia. The realization that a species is not static and that there is a wide range of variation within a species dates back to the mid-eighteenth century [137,138]. The core mechanisms by which species evolved were laid out by Darwin, who also popularized the origins of agriculture debates for the first time [64,139]. The unification of Darwinian evolutionary theory and genetics under the *modern synthesis* [140] brought with it the Biological Species Concept [141–143], fixated on reproductively isolated populations and genetic incompatibility. This species concept has many loopholes, as many ‘species’ are genetically compatible with phenotypically different populations that are ecologically isolated in the wild, what are often called ecotypes. The Ecological Species Concept [144] recognizes ecological isolation, focusing on behavioural, ecological and physiological traits. Morphologists (including palaeontologists) and ecologists tend to favour some version of the ecological species concept, whereas geneticists tend to lean towards the biological species concept. Ultimately, most taxonomists use some combination of these ideas, sometimes placed under the umbrella of an Evolutionary History Species Concept or the Phylogenetic Species Concept, as in modern cladistics. One open-ended definition is: ‘A species is any lineage of organisms that is distinct from other lineages because of differences in some shared biological property’ [84, p. 18].

Fitting the domestication concept into the species concept debates, one major feature stands out—a domesticated organism is rarely genetically isolated from its wild relatives. The realization that the basic biological parameters of the species concept are insufficient to define a domestication concept has been recognized at least since Harlan & de Wet [10] attempted to construct their own domestication concept. In this way, the biological concept does not apply as well as the ecological concept; therefore, when constructing a definition, an emphasis on phenotypic change should remain essential (as many scholars have stated in the past, e.g. [37,145]). Additionally, a focus on changes in allele frequencies as opposed to observable phenotypic traits opens up a can of worms, as nearly all human behaviours change the allele frequencies of organisms around them. For example, the process of logging a forest will change the allele frequencies among all remaining populations of trees within that forest. Hypothetically, every time a farmer subsamples their harvest and sets aside a portion of the seed to replant the next year or culls a herd, they are changing the allele frequencies of the remaining population [143,146,147]. Harlan & de Wet [10] follow some version of this concept, proposing a gene pool system, whereas a domesticated species includes all genetically compatible wild relatives, the ancestral population and distantly related populations that are still genetically compatible. In practice, this means that most domesticated populations are subspecies or varieties of a broader population that comprises both wild and domesticated morphs; the use of this system has become increasingly commonplace with the genetics revolution in taxonomy. Furthermore, within this phenotypic criterion, it must be recognized that no species can reverse-evolve or in this case become de-domesticated; therefore feral populations that behave or look phenotypically wild are still domesticated. These populations can pose a problem to the identification of domesticated organisms, especially in the case of crop-to-wild geneflow or hybridization.

Keeping in mind the need to emphasize heritable phenotypic traits, we can take Wilkins’s definition, which we just cited above, and modify it to represent a domesticated organism: *A domesticated clade is any lineage of organism that is phenotypically distinct from other lineages because of evolutionary differences resulting from interactions with humans*. In the same way, the most widely used definition of evolution (a change in allele frequencies (or heritable phenotypic traits) in a population over time) can be modified to define domestication: *A change in heritable phenotypic traits in a population over time resulting from interactions with humans* [148,149]. We favour these definitions, as they do not bias towards any specific preconceived ideas of what the earliest steps in the domestication process looked like and can be used by archaeologists and geneticists alike. Additionally, we favour avoiding the use of the term domestication in an active sense in scientific scholarship, suggesting instead that scholars discuss the evolution of domestication traits. For example, a correct usage would be: wheat evolved traits of domestication in response to cultural practices by ancient populations; an incorrect usage would be: ancient people domesticated wheat.

### (c) What does this concept include?

To clarify the parameters of the domestication guild, as defined here, we include all organisms that evolve in response to human activity, including all organisms evolving in response to mutualistic, commensal or parasitic relationships with humans, as well as predation (hunting) and anthropogenic habitat modification, including the introduction of toxins, such as herbicides. To reiterate, commensal domesticates are any organisms that evolve to have reduced stress responses in the presence of humans without benefitting or harming people; this includes the earliest steps in the domestication of the cat and dog, as well as mice and rats. This definition also includes birdfeeder birds and urban birds that express heritable endocrine changes in response to life in close proximity to humans [93,150–154]. Birds have also been recorded evolving to change their behaviours and morphology in response to urban noise and light pollution as well as to cars [88,91–93,155–157]. Urban foxes are currently in a process of evolving to become commensal domesticates, as European cities adopt canine extermination and sterilization programmes [98]. Studies of urban foxes show that they are evolving to change their morphology and behaviour in order to take advantage of the dump-heap-forager niche [158].

Our proposed domestication concept is also not limited by indicator alleles or specific domestication genes. In this way, a reduction in neural crest cell production can be domestication whether it has resulted from a deletion of segments of chromosome 7 (as supported by proponents of the William’s syndrome link [128]), transcriptional control through a regulatory or gatekeeper ‘Fox’ gene [159] or a domestication or candidate gene associated with neural crest cell production [160,161]. Furthermore, it is still domestication even if the neural crest cell hypothesis falls short of explaining what is going on [162]. The movement of a transposable element can lead to domestication, as in the evolution of the red hue in an apple [163] or colour change in the peppered moth [164]. Likewise, epigenetic factors, such as methylation, can result in domestication, as long as they are heritable and stable over many generations, such as in the domestication of the chicken [165] or dog [166], or in the methylation of a dominant receptor gene changing the behaviours of populations of great tits in response to continual exposure to humans [167]. The processes leading to domestication can be slow and protracted, as has been shown for many grain crops, or it can be rapid, as is the case of hybridization in many long-generation perennials. Additionally, given that the genes associated with domestication are most often present in the wild population before human intervention (as in the apple hue or peppered moth larval colour), domestication should not be thought of as necessarily associated with a new mutation or a novel allele, but rather the increase in prominence of one morphotype across a population. In most cases, the variation that evolution selects from under anthropogenic influence is already present in the wild, as in dimorphic seed-dispersal traits (e.g. tough rachises).

One criticism of the open-ended definition of domestication may be that it loses its utility if it is too broadly applied. Similar critiques have been made of open-ended definitions of Niche Construction Theory, which, in some iterations, becomes so loose in its application that it encompasses all biological processes (see discussion in [61]). We recognize the concern here, as some scholars may feel an emotional difference between a peppered moth and a dog, but our goal is to construct a definition based on logic as opposed to emotion. It is true that, as humanity moves into the modern era, the number of species that would fall under this concept of domestication will continue to rapidly increase. However, we see this as an analytical advantage, especially for conservation discussions. While some scholars would seek to limit the concept to only the first changes that happened in crops under early cultivation, expanding the definition to include all anthropogenic evolution illustrates the magnitude of human impacts on the planet. Humanity is responsible for a trajectory that will soon lead to all life being domesticated—the domestication of Earth.

## 4. Conclusions

Domestication studies remain one of the most prominent areas of research among archaeologists and are growing in prominence in other fields, including genetics. As the research purview expands, it becomes even more important that all scholars agree upon a clear definition. At the same time, many of the researchers in this area of study are changing their views regarding what the earliest steps in the domestication process would have looked like—Fuller has referred to this as a paradigm shift [3]. Given that not all scholars are in agreement, we seek consensus upon definitions that are inclusive and can encompass many differing views. Furthermore, a growing number of researchers have sought to attach ideological or moral trappings onto the domestication concept; some of these scholars claim that the ways the term is used are colonial legacies, implying that anyone still adhering to views from prior to the 2009 paradigm shift is propagating colonialist ideologies [50]. Other scholars latch onto the failure-to-domesticate paradox; for these, any ancient peoples that did not drive significant evolutionary change in the organisms around them must have been less intelligent. These scholars latch onto concepts such as anti-domestication or landscape domestication in order to empower the legacy of those ancient peoples. Given that all of these ideological or moralistic trends arise out of very distinct ideas of what domestication looked like, notably that it was rapid and the result of great human ingenuity and control over nature, it is especially important that we develop a definition that is divorced from any of these ideological agendas.

The open-ended definition of domestication that we push in this paper (*A change in heritable phenotypic traits in a population over time resulting from interactions with humans*) would suggest that organisms as widely differing as weeds in your garden, birds at your bird feeder, your pet cat and even yourself fall under the umbrella of domesticated. The expansive definition suggests that, as the planet moves into the modern era, it is undergoing a radiation and diversification of domesticated organisms that loosely parallels the angiosperm radiation. This would suggest that domesticated organisms or the organisms that have evolved to survive or even thrive in anthropogenic ecosystems, are on a trajectory towards global conquest. In the not-too-distant future, most of the life on Earth will be domesticated.

## Data Availability

Supplementary material is available online [168].
